# Short-Term and Long-Term Effects after Exposure to Ionizing Radiation and Visible Light on Retina and Retinal Pigment Epithelium of Mouse Eye

**DOI:** 10.3390/ijms242317049

**Published:** 2023-12-01

**Authors:** Tatiana Feldman, Marina Yakovleva, Dina Utina, Mikhail Ostrovsky

**Affiliations:** 1 Department of Biology, Lomonosov Moscow State University, Leninskiye Gory 1, Moscow 119234, Russia; ostrovsky3535@mail.ru; 2 Emanuel Institute of Biochemical Physics, Russian Academy of Sciences, 4 Kosygin Street, Moscow 119334, Russia; lina.invers@gmail.com; 3 Koltzov Institute of Developmental Biology, Russian Academy of Sciences, 26 Vavilov Street, Moscow 119334, Russia; 4 Laboratory of Radiation Biology, Joint Institute for Nuclear Research, Joliot-Curie 6, Dubna 141980, Russia; din-din-86@mail.ru

**Keywords:** eye, retina, retinal pigment epithelium, retinoid fluorophores, ionizing radiation, visible light, fluorescence analysis, HPLC

## Abstract

A comparative in vivo study of the effects of ionizing radiation (accelerated protons) and visible light (400–700 nm) on the retina and retinal pigment epithelium (RPE) of the mouse eye was carried out. Using the methods of fluorescence spectroscopy and high-performance liquid chromatography (HPLC), we analyzed the relative composition of retinoids in chloroform extracts obtained from the retinas and RPEs immediately after exposure of animals to various types of radiation and 4.5 months after they were exposed and maintained under standard conditions throughout the period. The fluorescent properties of chloroform extracts were shown to change upon exposure to various types of radiation. This fact indicates the accumulation of retinoid oxidation and degradation products in the retina and RPE. The data from fluorescence and HPLC analyses of retinoids indicate that when exposed to ionizing radiation, retinoid oxidation processes similar to photooxidation occur. Both ionizing radiation and high-intensity visible light have been shown to be characterized by long-term effects. The action of any type of radiation is assumed to activate the mechanism of enhanced reactive oxygen species production, resulting in a long-term damaging effect.

## 1. Introduction

Ionizing radiation (IR) causes pathological, functional and morphological changes in living organisms. IR induces cellular formation of free radicals and triggers the processes of protein destruction, DNA damage, development of malignant tumors, cell degeneration and potentially the ultimate death of the exposed organism [1,2,3,4,5,6,7]. It is well known that radiation-induced cell damage involves the generation of reactive oxygen species (ROS) and subsequent development of oxidative stress [8,9,10,11]. 

This work is devoted to a study of short-term and long-term effects after exposure to IR on the retina and retinal pigment epithelium (RPE) of the mouse eye. Also, these effects are compared to the damaging effects of visible light.

**Figure 1:**
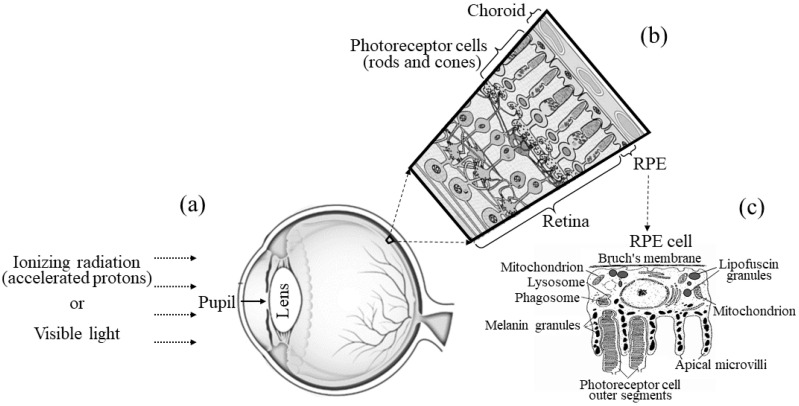
Scheme of the vertebrate eye, retina, retinal pigment epithelium (RPE) and the RPE cell. (**a**)—The effect of ionizing radiation (accelerated protons) and visible light on the eye structures: pupil, lens, retina and RPE; (**b**)—the retina and RPE structures: photoreceptor cells (rods and cones), RPE and choroid; (**c**)—RPE cell: Bruch’s membrane, mitochondrion, lysosome, phagosome, melanin granules, photoreceptor cell outer segments, apical microvilli and lipofuscin granules containing retinoid derivatives. Figure was modified from [13].

The retina is the light-sensitive tissue of the eye (Figure 1a). It consists of several layers of neurons interconnected by synapses (Figure 1b). The primary light-sensing cells of the retina are the photoreceptor cells, the rods and cones. The RPE is the pigmented single-cell layer located just behind the retina, firmly attached to the underlying choroid and is in close contact with photoreceptor cells (Figure 1b). The RPE has several crucial functions for vision, namely, scattered light absorption, epithelial transport, spatial ion buffering, visual cycle, phagocytosis of photoreceptor outer segment membranes, secretion and immune modulation [12]. 

According to morphological studies, the retina of adult animals is more radio-resistant compared to other tissues of the organism [14]. However, analysis of numerous human space flights has shown that the eye, along with the skin, is the most dose-accumulating organ [15]. Therefore, despite the relatively high radio-resistance of the retina, many astronauts experience early cataractogenesis and other visual pathologies [16].

To date, a number of studies have shown that IR can potentially induce retinal oxidative damage and apoptosis [17]. IR-exposure has been shown to induce oxidative changes in the retina and continuous remodeling of retinal microvessel architecture over the course of a year after irradiation [18,19,20]. Thus, oxidative stress is likely to be involved in the pathogenesis of radiation-induced retinal damage [21].

Our previous research revealed that various types of IR caused retinoid oxidation in the retina and RPE of the mouse eye [13]. The importance of the obtained results lies in the fact that oxidized retinoids are toxic to the retina and RPE cells [22,23,24]. The oxidation of retinoids leads to the formation of various products, consisting mainly of aldehydes and ketones [25,26,27,28]. Reactive carbonyls are known to be highly cytotoxic; they can modify cellular proteins and lipids and induce carbonyl stress [29,30]. Consequently, we can conclude that retinoid oxidation products have a damaging effect on the retina and RPE cells and can be considered as an aggravating factor in the progression of age-related macular degeneration. Radiation-induced oxidation of retinoids cause a significant change in their fluorescent properties, which allows us to consider this phenomenon as a potential opportunity for non-invasively assessing the degree of radiation exposure and its relative biological effect in humans. This assessment can potentially be facilitated by using an advanced noninvasive diagnostic method in ophthalmology, the spectral analysis [31,32,33] of fundus autofluorescence (FAF). This method is based on the fact that the relative content of retinoid photooxidation products from the RPE cells obtained from human cadaveric eyes increases with age and with pathology, however, only in the case of pathology is there a maximum shift of the fluorescence spectrum of the chloroform extract of RPE to the short-wavelength region. Thus, oxidized retinoids can be markers of radiation-induced pathological processes in the retina and RPE. 

The results of our studies on the effects of IR on mice [13] allowed us to assume that the oxidation of retinoids occurs similarly to their photooxidation. Thus, the purpose of this work was to conduct a comparative study of the effects of various types of radiation (visible light and accelerated protons) on the retina and RPE of the mouse eye to confirm this assumption. In addition, a study was conducted on the short- and long-term effects after exposure to IR and visible light on the retina and RPE of the mouse eye.

## 2. Results

**Figure 2:**
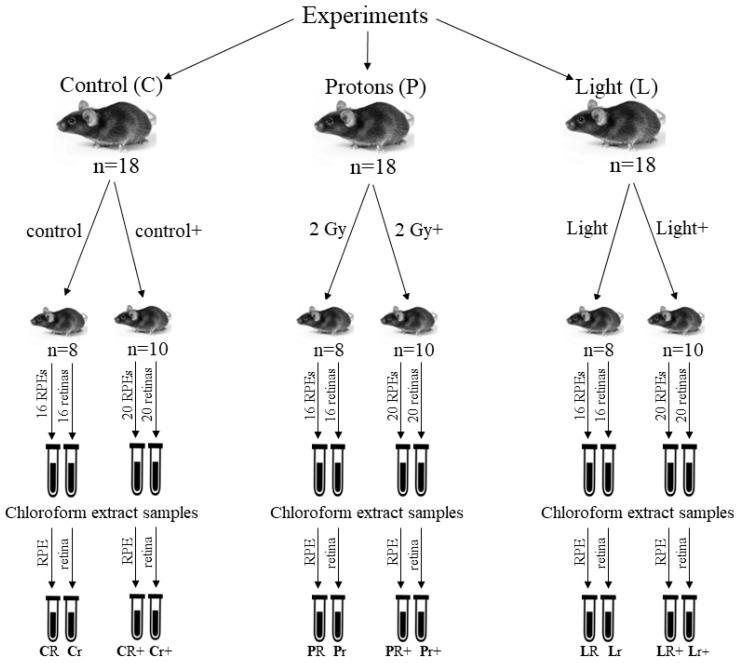
Scheme of experiments on the effects of ionizing radiation (Protons) and visible light (Light) on the retina and RPE of the mouse eye. Control (C)—a group of control animals; Protons (P)—mice irradiated with accelerated protons at a dose of 2 Gy; Light (L)—mice irradiated with visible light (400–700 nm) with a luminous flux of 13,000 lux for 2 h. R—RPE samples, r—retina samples, +—samples obtained 4.5 months after irradiation of mice.

We performed comparative in vivo study on the effects of various types of radiation (visible light and accelerated protons) on the mouse eye. Since the experiments on the irradiation of animals with accelerated protons and visible light were carried out simultaneously, in both cases, common control samples of chloroform extracts of the retinas and RPEs from the eyes of mice that were not exposed to any irradiation were used. Figure 2 shows the scheme of experiments. Before and after the experiments, all animals were kept in the vivarium under the same standard conditions. It should be noted that during the entire study, both immediately after irradiation and after 4.5 months of keeping the mice in the vivarium, all animals remained alive.

**Figure 3:**
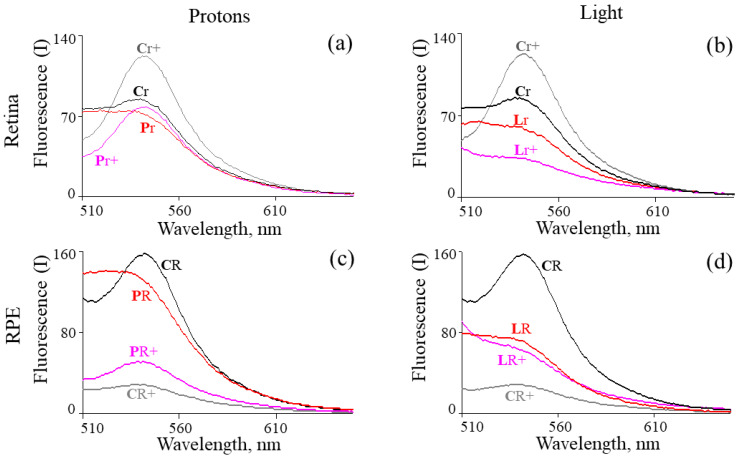
Fluorescence spectra of chloroform extracts of retinas and RPEs from mouse eyes (*n* = 8 or 10/group) with accelerated protons at a dose of 2 Gy or visible light (400–700 nm) exposure. (**a**)—Samples from the retinas of mice irradiated with accelerated protons; (**b**)—samples from the retinas of mice irradiated with visible light; (**c**)—samples from the RPE of mice irradiated with accelerated protons; (**d**)—samples from the RPE of mice, irradiated with visible light. Designations: C—control animals (black spectra), P—accelerated proton irradiation (red spectra), L—visible light irradiation (red spectra); R—RPE samples, r—retina samples, +—samples obtained 4.5 months after irradiation of mice (gray spectra for control samples; rose spectra for irradiated samples). The excitation wavelength was 488 nm. The designations of the samples correspond to the name of the samples in Figure 2.

Figure 3 shows the fluorescence spectra of chloroform extracts from the retinas and RPEs of mouse eyes. It can be seen from the figure that after exposure to various types of radiation, the shape and intensity of the fluorescence spectra of the studied samples, compared to the control, change both in the samples obtained one day after irradiation and 4.5 months later. It should be noted that fluorescence analysis does not allow to obtain absolute quantitative characteristics of changes in the studied samples.

On the one hand, the age of the animals more than doubled 4.5 months after irradiation, and, accordingly, the relative content of retinoids could also have changed regardless of the experiment. This assumption was confirmed by the peculiarities of changes in fluorescence characteristics of chloroform extracts of the control samples. In the case of a retinal control sample obtained 4.5 months after keeping the animals under standard conditions (Cr+), the fluorescence intensity increased (approximately 1.5-fold with a maximum of 554 nm) compared to the original control sample (Cr) (Figure 3a,b). In the case of the RPE control sample obtained after 4.5 months (CR+), the fluorescence intensity in the maximum region (554 nm) decreased markedly (by about 6 times) compared to the original control sample (CR) (Figure 3c,d). Such a change in the fluorescent properties of chloroform extracts of the retinas and RPEs, depending on the age of animals, can be explained by a change in the relative composition of retinoids with age. This assumption correlates well with our research results [25], which showed that the ratio of bisretinoid photo-oxidation and photodegradation products to unoxidized bisretinoids in the chloroform extract of cadaver-eye RPE increases with donor age, from 0.69 ± 0.03 to 1.32 ± 0.04. Besides, in the work [34], in vivo images were obtained from mice between the ages of 2 and 32 months. The cSLO (confocal scanning laser ophthalmoscope) images showed an increase in auto-fluorescence at the photoreceptor-RPE interface as age increases. 

On the other hand, it was impossible to conduct several similar experiments to collect statistics. In this regard, the previously proposed approach was used to quantify changes in the relative content of oxidized forms of retinoids [31]. In this study, we evaluated the dynamics of changes in the relative content of non-oxidized and oxidized forms of retinoids when animals were irradiated with visible light or IR by the ratio of the fluorescence intensity of chloroform extract samples at a wavelength of 522 nm to values at a wavelength of 554 nm (522/554 nm). The Table 1 shows that for all samples (retina and RPE) obtained immediately after irradiation of animals (short-term effect), both with visible light and accelerated protons, an increase in the ratio of fluorescence intensity values was observed compared with control samples. These results indicate that exposure to any type of radiation leads to an increase in the relative content of oxidized forms of retinoids [25,31]. It should be noted that there is no noticeable difference in the values obtained, both for light-irradiated animals and those irradiated with accelerated protons. 

A different pattern is observed for samples obtained 4.5 months after irradiation of animals (long-term effect). In an experiment with visible light irradiation of animals, an increase in oxidized forms of retinoids was observed in samples of chloroform extracts from retinas and RPEs, similar to the short-term effect. However, after exposure to accelerated protons (long-term effect), the numerical indicator decreased and became close to the control values (Table 1). This result suggests that 4.5 months after exposure to IR, the initial balance of oxidized and non-oxidized retinoids, corresponding to the norm, was restored in the retina and RPE. Fluorescence analysis, however, could not give a definite answer about the correctness of such a conclusion, since more than 20 fluorophores were present in the chloroform extract, some of which had similar spectral properties [35]. In addition, the spectral properties of highly oxidized retinoids are characterized by absorption in the UV region. At the same time, they can be chemically active carbonyl compounds that can interact with biological molecules [22,23]. In such a situation, these products can also affect the spectral properties of the studied samples.

**Figure 4:**
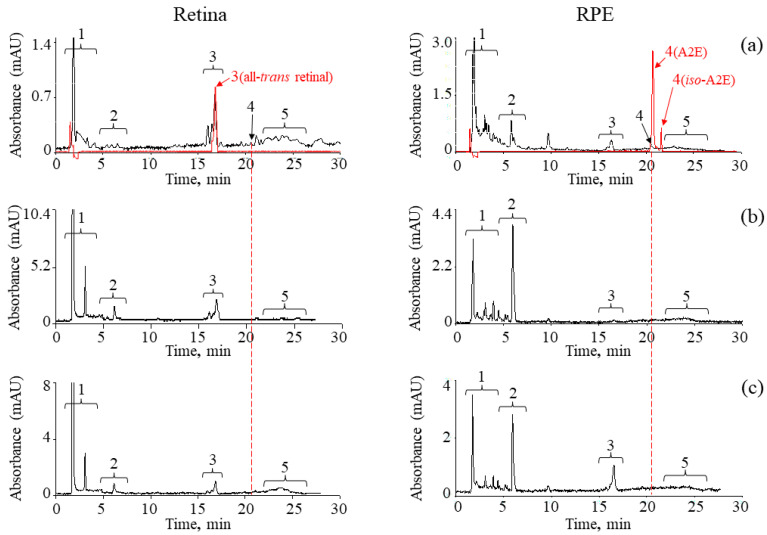
Chromatograms of chloroform extracts of retinas and RPEs from eyes of mice without (controls) (**a**) and with exposure to accelerated protons (**b**) or visible light (**c**) obtained immediately after irradiation (short-term effect). Peaks/groups of peaks on the chromatogram: 1, 2—oxidized forms of retinoids [25,31]; 3—retinal isomers; 4—bisretinoid A2E; 5—retinoids (bisretinoids) and oxidized forms of retinoids [35,36]; A2E and all-*trans* retinal standards are represented by red chromatograms. Absorption detection was monitored at 430 nm.

To understand whether the balance of oxidized and non-oxidized retinoids in the retina and RPE of the mouse eye is indeed restored 4.5 months after irradiation of animals with accelerated protons, we carried out HPLC analysis of all chloroform extract samples. Figure 4 shows chromatograms of chloroform extracts of retinas and RPEs from eyes of mice without and with exposure to accelerated protons or visible light obtained immediately after irradiation (short-term effect). 

**Figure 5:**
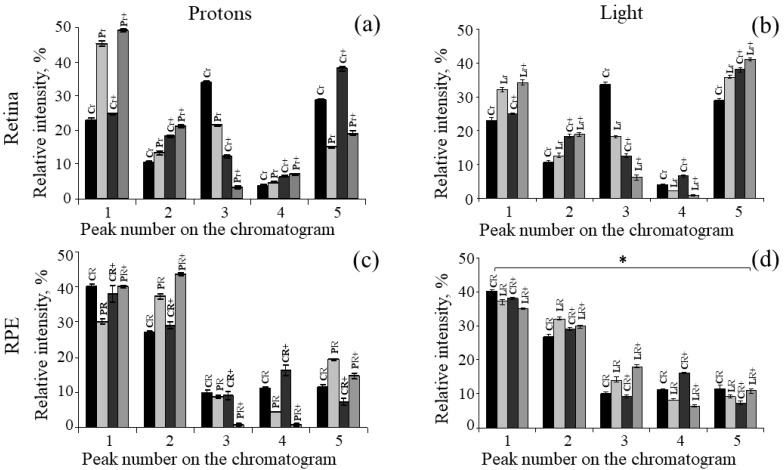
Diagrams of the relative content of various retinoids and their derivatives in chloroform extracts of retinas and RPEs from eyes of mice with and without exposure to accelerated protons at doses of 2 Gy or visible light (400–700 nm). On the abscissa axis, the numbers of peak groups correspond to the designations in Figure 4. On the ordinate axis, the relative content of the corresponding peak groups is plotted as a percentage. (**a**)—Samples from the retinas of mice irradiated with accelerated protons; (**b**)—samples from the retinas of mice irradiated with visible light; (**c**)—samples from the RPE of mice irradiated with accelerated protons; (**d**)—samples from the RPE of mice, irradiated with visible light. Designations: C—control animals, P—accelerated proton irradiation, L—visible light irradiation; R—RPE samples, r—retina samples, +—samples obtained 4.5 months after irradiation of mice. The designations above the columns correspond to the designations in Figure 2 and Figure 3. * Data are shown as mean ± SD from three independent HPLC experiments conducted for each type of radiation, *p* < 0.05.

On the basis of chromatographic analysis, we evaluated the dynamics of changes in the relative content of non-oxidized and oxidized forms of retinoids in the retina and RPE during irradiation of animals with light or IR compared with control animals (Figure 5). Previously, we studied in detail the products of photooxidation of synthetic A2E and lipofuscin granule bisretinoids from the RPE of human cadaveric eyes using HPLC analysis [25,31]. A set of similar products was observed in the retina and RPE of the mouse eye. One can only note the difference in the lower content of bisretinoid A2E in the mouse eye RPE compared to the human RPE. For a detailed analysis, we considered separately the trends in the relative content of different groups of retinoids in the retina and RPE after irradiation of mice with accelerated protons and visible light immediately after exposure (short-term effect) and after 4.5 months (long-term effect).

The retina. Comparative HPLC analysis of the dynamics of changes in the relative content of oxidized forms of retinoids (Figure 5a,b: group of peaks 1; Table 2 and Table 3) showed that both exposure to accelerated protons and visible light resulted in a noticeable increase (short-term effect) in the relative content of oxidized forms of retinoids included in the group of peaks 1 compared with the control. At the same time, it was noted that in the case of the experiment with accelerated protons, the content of oxidized forms of retinoids increased by more than 20% compared to the experiment on irradiation of animals with visible light (about 10%). For groups of peaks 2 (oxidized forms of retinoids) and 3 (retinal isomers: 11-*cis*, all-*trans*), it can be concluded that the dynamics of changes in the relative content of these compounds in both experiments were identical. The relative content of retinal isomers decreased especially noticeably compared to the control. For the group of peaks 5 [35,36], the opposite trend was observed in the dynamics of changes in the relative content of these compounds in different experiments. In the experiment with accelerated protons, there was a noticeable (almost 2-fold) decrease in the relative content of compounds in the group of peaks 5 (Figure 5a: group of peaks 5; Table 2). In an experiment with visible light irradiation, on the contrary, a slight increase was observed (Figure 5b: group of peaks 5; Table 3).

Thus, a comparative study of the dynamics of changes in the relative content of non-oxidized and oxidized retinoids in the mouse retina showed that the effect of accelerated protons on the retina caused a more pronounced effect in increasing the relative content of oxidized forms of retinoids compared with control samples. As for the compounds included in the group of peaks 5, it can be assumed that the effect of accelerated protons led to a greater degree of oxidation and degradation of these compounds, which, in turn, may have led to a decrease in the retention time of these compounds during HPLC analysis. It is likely that the more pronounced effect of changes in the relative content of compounds in the group of peaks 1 under accelerated proton irradiation was a consequence of the oxidation of compounds in the group of peaks 5. 

A comparison of the results of experiments on short-term and long-term effects showed that the dynamics of an increase in the relative content of oxidized forms of retinoids in the retina persisted 4.5 months after exposure to mice with both visible light and ionizing radiation (Figure 5a,b, group of peaks 1; Table 2 and Table 3). In addition, the relative content of retinal isomers (peak group 3) after 4.5 months became noticeably less compared to the control in both experiments, and the relative content of compounds from the group of peaks 5 in the long-term effect retained the dynamics of change similar to the short-term effect. In other words, the processes of restoring the balance of oxidized and non-oxidized retinoids in the retina, characteristic of the norm, did not occur over a sufficiently long-time interval (4.5 months).

The RPE. In contrast to the samples obtained from the retinas, the RPE samples initially showed a higher (about twofold) relative content of oxidized retinoids corresponding to groups of peaks 1 and 2 on the chromatogram (Figure 5c,d). More significant changes after irradiation could be noted in the relative content of oxidized forms of retinoids from the group of peaks 2 (Figure 5c,d: group of peaks 2; Table 2 and Table 3). At the same time, a more pronounced effect was observed after exposure to accelerated protons. For the remaining compounds from the groups of peaks 1, 3, 4 and 5, the dynamics of changes in their content was also observed. A decrease in the A2E content (peak 4) could be noted after exposure to both light and accelerated protons, which may indicate A2E oxidation process [25]. 

Comparison of the results of experiments on short-term and long-term effects showed a similar dynamic of changes in the relative content of all components in chloroform extracts obtained from the RPE of mouse eyes after exposure to visible light or IR. This allows us to assume that the processes of oxidation and degradation of retinoids in the RPE, similarly to the retina, have a prolonged character. 

## 3. Discussion

This paper presents the results of an in vivo comparative study of the effects of IR (accelerated protons) and visible light (400–700 nm) on the mouse eye. We analyzed the dynamics of changes in the relative content of non-oxidized and oxidized retinoids in the retina and RPE of the mouse eye.

Previously, we had already conducted studies of the effect of IR (accelerated protons and gamma rays) on the retina and RPE of the mouse eye [13]. It has been shown that under the action of IR (short-term effect), retinoid oxidation occurs in these tissues. It was assumed that the oxidation process induced by IR is similar to photo-oxidation. IR is known to cause the formation of reactive oxygen species (ROS) and subsequent oxidative stress [11,37,38,39,40]. It is possible that IR-induced ROS can also initiate retinoid oxidation in the absence of light.

In this investigation, a comparative study of the effects of IR and visible light confirmed this assumption. Moreover, in contrast to previous studies [13], the long-term effect after exposure to both types of radiation on the mouse eye was also studied. HPLC analysis of retina and RPE chloroform extracts obtained 4.5 months after animal exposure to accelerated protons and visible light (long-term effect) was performed. The results obtained in the long-term effect were comparable to the short-term effect when exposed to both types of radiation. Naturally, the following question arises: what mechanism of oxidation of retinoids in the tissues of the eye is triggered after exposure to radiation?

It is known that in vertebrates throughout life, the debris of the photoreceptor outer segment apical part (Figure 1c), are phagocytized and digested by the RPE cells, while the new photoreceptor discs with rhodopsin molecules are synthesized by the photoreceptor inner segments [41]. Given this fact, it could be assumed that over time we should observe a decrease in the relative content of oxidized forms of retinoids in the retina and a restoration of the balance of non-oxidized and oxidized retinoids characteristic of the norm. However, the results of HPLC analysis show that the long-term effect after exposure to various types of radiation, especially ionizing, manifests itself to the same extent as the short-term effect. This fact allows us to assume that after exposure to radiation (both ionizing and visible light), the mechanisms of generation of ROS with a prolonged effect are activated in the photoreceptor cell, leading to the continuation of retinoid oxidation in the retina. A similar phenomenon, for example, is well known in the field of radiobiological research [11,37,38,39,40]. Thus, the effect of IR on a living organism causes increased formation of ROS with a prolonging effect [37], which, in turn, leads to the development of oxidative stress, and subsequent cell death. It is believed that mitochondrial dysfunction caused by ionizing radiation is one of the main sources of an increase in intracellular ROS level [11]. Thus, the results of this study give grounds to conclude that similar processes of enhanced ROS formation with a prolonging effect may also occur in retinal photoreceptor cells, which, in turn, lead to an increase in the level of oxidized forms of retinoids. It can be assumed that similar processes are observed in RPE cells.

Our assumption correlates well with the results of studies where it was shown that the extreme radiosensitivity of the photoreceptor cells was underlined by the continued manifestation of fine structural changes and cell death up to 6 months post-X-ray-radiation in animals receiving doses above 500 cGy [42]. Moreover, in the works [17,18,19,20], it was shown that IR-exposure causes oxidative changes in the retina over the course of a year after irradiation.

Notably, a similar phenomenon was observed in our experiments when the retina and RPE were exposed to high-intensity visible light. That is, the results of the experiments allow us to conclude that the process of photodamage is also characterized by a prolonged effect that occurs as a result of the launch of the intracellular mechanism, leading to an increase in the content of intracellular ROS level. It should be noted that the mechanisms of retina and RPE photodamage have been studied quite well [43,44]. However, our work presents the results of the effects of visible light exposure after 4.5 months (long-term effect). These results allow for a deeper understanding of the mechanisms of visual pathology occurrence under the action of high-intensity visible light. 

## 4. Materials and Methods

### 4.1. Reagents

All reagents were purchased from Sigma-Aldrich (St. Louis, MO, USA) and Fluka (Buchs, Switzerland). 

### 4.2. Animals

For the experiments, we used male mice, hybrids of the first generation C57BL/6 × CBA at the age of 6 months, with an average weight of 31 g. The animals were purchased at the Laboratory Animal Nursery Branch of the Shemyakin-Ovchinnikov Institute of Bioorganic Chemistry of the Russian Academy of Sciences (Moscow region, Pushino, Russia). 

### 4.3. Irradiation of Mice with Accelerated Protons

**Figure 6:**
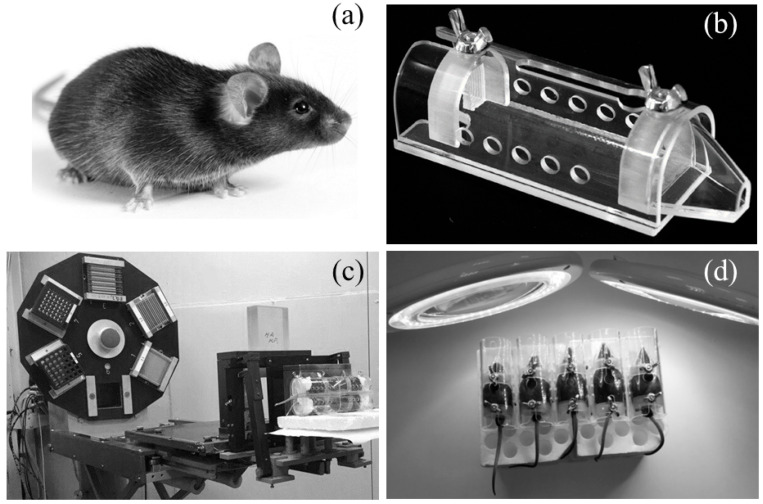
In vivo experiments with mice. (**a**)—A male mouse, a hybrid of the first generation C57BL/6 × CBA at the age of 6 months, weighing 31 g; (**b**)—individual holder used to keep non-anesthetized mice in a stationary position during irradiation; (**c**)—irradiation of mice with accelerated protons on a medical beam of the JINR phasotron (Dubna, Russia); (**d**)—irradiation of mice with visible light (400–700 nm) with a luminous flux of 13,000 lux for 2 h.

The heads of mice were irradiated with accelerated protons using a medical phasotron beam of the Joint Institute for Nuclear Research (JINR, Moscow Region, Dubna) with frequency modulation and a 70 mm collimator (Figure 6a–c). The average proton energy of the chamber inlet upstream of the decelerator was 170 MeV. The energy of the particles in the chamber was determined by the beam range in water (R = 200 mm). Measurements were carried out with a silicon semiconductor detector. The average value of the LET at the beam inlet was 0.49 keV/µm [45]. Dosimetric beam calibration at each point of the deep dose distribution was carried out by the TM30013 ionization chamber of the PTW UNIDOS-E clinical dosimeter. Irradiation of animals was carried out at a dose of 2 Gy. The animals were irradiated in special individual fixators to keep the mice in a stationary state (4 individuals at a time). The holders were made of transparent acrylic plastic (plexiglass) measuring 90 × 30 × 26 mm and had a cylindrical shape with two movable limiters on both sides. 

### 4.4. Irradiation of Mice with Visible Light

The animals were irradiated with visible light (400–700 nm) with a luminous flux of 13,000 lux (luxmeter Testo-AG, Titisee-Neustadt, Germany) for 2 h (Figure 6d). A day after the irradiation, a subset of the animals was sacrificed by the method of cervical dislocation, followed by the removal of the eyes, from which the retinas and RPEs were then extracted separately, as described in [46]. All extractions and manipulations with tissues were carried out on ice. The remaining mice after irradiation were kept in a vivarium under standard conditions for 4.5 months, after which they were also killed, and retinas and RPEs were extracted from the eyes. 

The animals were not anesthetized during irradiation.

### 4.5. Fluorescent Analysis of Chloroform Extracts of Retinoids from the Retina and RPE

Chloroform extracts of retinoids from retina and RPE suspensions were obtained by the Folch method [47]. In short, a twofold excess of the chloroform: methanol (2:1 vol./vol.) mixture was added to each sample of the RPE or retina suspension. The samples were stirred using an electric stirrer (ELMI laboratory technology, Latvia) for 2 min, followed by incubation for 10 min at 48 °C. Each mixture was centrifuged at 680.3× *g* (MLW K26 D, EB Zentrifugenbau Engelsdorf, Hamburg, Germany) for 10 min at 48 °C. The lower layer containing chloroform extract was taken with a syringe and transferred to a flask.

Fluorescence spectra of chloroform extracts were recorded by an RF-5301 PC spectrofluorometer (Shimazu, Tokyo, Japan) equipped with an R955 lamp photomultiplier detector (Hamamatsu, Hamamatsu, Japan) using RFPC software version 2.0 (Shimazu, Japan). Fluorescence excitation was carried out with a wavelength of 488 nm, similar to the diagnostic method in ophthalmology—fundus autofluorescence [48]. Taking into account the intensity of the excitation, the fluorescence spectra were corrected using the spectral response (quantum efficiency) of a lamp detector with a photomultiplier R955. 

All stages of sample preparation were carried out under dim red lighting.

### 4.6. High Performance Liquid Chromatography (HPLC)

For HPLC analysis, chloroform extracts were evaporated using a vacuum pump (Vacuubrand MZ 2CNT+AK+M+D, Wertheim, Germany). The dried samples were suspended in 200 µL of methanol. Composition of retinoids were analyzed using chromatographic equipment (Knauer, Berlin, Germany) with a 110 C18 Diasfer column (4 × 250 mm; sorbent size 5 microns). Separation was carried out by linear gradient elution from 80% acetonitrile/20% water (vol./vol.) (+0.05% TFC) to 100% acetonitrile for 20 min at an elution rate of 1.0 mL/min. Absorption (K-2501 detector, Knauer) was measured at 430 nm. Since some retinoid fractions are detected at the early stages of retention, the solvent peak (front) was removed from the chromatogram to exclude its contribution. The absorption efficiency was evaluated in the form of peak areas (mV × s) using the EuroChrom 5.05 chromatographic program. The measurement accuracy was determined based on three independently measured chromatograms for each individual sample. The surface area of each component in the mixture was calculated as a percentage of the total surface area of all peaks, including the relative content of each component. Most of the detectable products are unknown, so their relative content has been estimated independently of their extinction coefficients. The significance of the differences between the groups (*p*) was calculated using the Student’s criterion.

Bisretinoid A2E synthesized according to the method described in [49], as well as all-*trans* retinal by Sigma-Aldrich (USA) were used as standards. 

All stages of sample preparation were carried out under dim red lighting. 

### 4.7. Statistical Analysis

The results obtained from all experiments were analyzed by one-way analysis of variance (ANOVA). The significance difference was set at *p* < 0.05. Data are shown as mean ± standard deviation (SD). 

## 5. Conclusions

The work was devoted to the study of short- and long-term effects after exposure to IR and visible light on the retina and RPE of the mouse eye. Fluorescent and HPLC analyses of the relative content of non-oxidized and oxidized retinoids in the eye tissues revealed the dynamics of accumulation of retinoid oxidized forms not only immediately after exposure to both types of radiation but also after a sufficiently long period after exposure. 

Since oxidized forms of retinoids are an indicator of destructive processes in the retina and RPE cells, we can conclude that the damaging effect of both types of radiation is manifested not only at the time of its impact, but the result of their influence is characterized by a prolonged effect. It is supposed that the action of any type of radiation activates the mechanisms of enhanced ROS production, resulting in such long-term damaging effects.

The results obtained can be used to develop a preclinical diagnostic method in ophthalmology—spectral analysis of the relative content of non-oxidized and oxidized retinoids suggested by us [31], based on the principles of non-invasive diagnosis of degenerative processes in the retina and RPE—FAF [48].

## Figures and Tables

**Table 1:** The ratio of fluorescence intensity values (522/554 nm) of chloroform extracts obtained from the retinas and RPEs of mice eyes after irradiation of animals with accelerated protons or visible light. The wavelength of fluorescence excitation is 488 nm *.

Chloroform Extract Samples from the Retinas			
Control Cr	Protons, 2 Gy Pr	Control (after 4.5 months) Cr+	Protons, 2 Gy (after 4.5 months) Pr+
1.13	1.26	0.70	0.82
Control Cr	Light Lr	Control (after 4.5 months) Cr+	Light (after 4.5 months) Lr+
1.13	1.30	0.70	1.30
**Chloroform Extract Samples from the RPEs**			
Control CR	Protons, 2 Gy PR	Control (after 4.5 months) CR+	Protons, 2 Gy (after 4.5 months) PR+
0.91	1.37	1.10	0.97
Control CR	Light LR	Control (after 4.5 months) CR+	Light (after 4.5 months) LR+
0.91	1.39	1.10	1.49

* All designations correspond to the designations in Figure 3.

**Table 2:** HPLC analysis of the relative content of retinoids and their oxidized forms in the retina and RPE of mouse eyes before and after exposure to accelerated protons.

Chloroform Extract Samples from the Retinas				
№ Peak/Group of Peaks *	Control Cr %	Protons, 2 Gy Pr %	Control (after 4.5 Months) Cr+ %	Protons, 2 Gy (after 4.5 Months) Pr+ %
1	22.9 ± 0.8	45.3 ± 0.7	24.8 ± 0.2	49.1 ± 0.3
2	10.5 ± 0.5	13.4 ± 0.7	18.3 ± 0.5	21.3 ± 0.5
3	33.7 ± 0.8	21.4 ± 0.4	12.4 ± 0.6	3.3 ± 0.5
4	4.0 ± 0.2	4.8 ± 0.1	6.5 ± 0.2	7.1 ± 0.3
5	28.9 ± 0.5	15.1 ± 0.3	38.0 ± 0.8	19.2 ± 0.7
**Chloroform Extract Samples from the RPEs**				
**№ Peak/Group of Peaks** *	**Control** **CR** **%**	**Protons, 2 Gy** **PR** **%**	**Control** **(after 4.5 Months)** **CR+** **%**	**Protons, 2 Gy** **(after 4.5 Months)** **PR+** **%**
1	40.1 ± 0.8	30.2 ± 0.6	38.1 ± 1.3	40.1 ± 0.3
2	26.8 ± 0.5	37.2 ± 0.7	29.1 ± 0.9	43.7 ± 0.5
3	10.1 ± 0.8	8.9 ± 0.4	9.2 ± 0.4	0.7 ± 0.5
4	11.3 ± 0.2	4.4 ± 0.1	16.3 ± 0.6	0.8 ± 0.3
5	11.7 ± 0.5	19.3 ± 0.3	7.3 ± 0.7	14.7 ± 0.7

* The peak numbers correspond to the designations in Figure 4.

**Table 3:** HPLC analysis of the relative content of retinoids and their oxidized forms in the retina and RPE of mouse eyes before and after exposure to visible light.

Chloroform Extract Samples from the Retinas				
№ Peak/Group of Peaks *	Control Cr %	Light Lr %	Control (after 4.5 Months) Cr+ %	Light (after 4.5 Months) Lr+ %
1	22.9 ± 0.8	32.1 ± 0.8	24.8 ± 0.2	34.2 ± 0.9
2	10.5 ± 0.5	12.5 ± 0.6	18.3 ± 0.5	18.7 ± 0.7
3	33.7 ± 0.8	18.2 ± 0.6	12.4 ± 0.6	5.2 ± 0.3
4	4.0 ± 0.2	2.1 ± 0.1	6.5 ± 0.2	0.8 ± 0.1
5	28.9 ± 0.5	35.1 ± 0.3	38.0 ± 0.8	41.1 ± 0.7
**Chloroform Extract Samples from the RPEs**				
**№ Peak/Group of Peaks** *	**Control** **CR** **%**	**Light** **LR** **%**	**Control** **(after 4.5 Months)** **CR+** **%**	**Light** **(after 4.5 Months)** **LR+** **%**
1	40.1 ± 0.8	37.1 ± 0.6	38.1 ± 1.3	35.1 ± 0.7
2	26.8 ± 0.5	32.1 ± 0.9	29.1 ± 0.9	29.8 ± 0.8
3	10.1 ± 0.8	13.3 ± 0.5	9.2 ± 0.4	17.7 ± 0.6
4	11.3 ± 0.2	8.3 ± 0.2	16.3 ± 0.6	6.5 ± 0.4
5	11.7 ± 0.5	9.2 ± 0.3	7.3 ± 0.7	10.9 ± 0.5

* The peak numbers correspond to the designations in Figure 4.

## Data Availability

The data presented in this study are available in the article and on request from the corresponding author.

## References

[1] Kennedy A.R. (2014). Biological effects of space radiation and development of effective countermeasures. Life Sci. Space Res..

[2] Sanzari J.K., Wan X.S., Wroe A.J., Rightnar S., Cengel K.A., Diffenderfer E.S., Krigsfeld G.S., Gridley D.S., Kennedy A.R. (2013). Acute hematological effects of solar particle event proton radiation in the porcine model. Radiat. Res..

[3] Finnberg N., Wambi C., Kennedy A.R., El-Deiry W.S. (2013). The effects of antioxidants on gene expression following gamma-radiation (GR) and proton radiation (PR) in mice in vivo. Cell Cycle.

[4] Kennedy A.R. (2006). Effects of dietary supplements on the space radiation-induced reduction in total antioxidant status in CBA mice. Radiat. Res..

[5] Kennedy E.M., Powell D.R., Li Z., Bell J.S.K., Barwick B.G., Feng H., McCrary M.R., Dwivedi B., Kowalski J., Dynan W.S. (2018). Galactic cosmic radiation induces persistent epigenome alterations relevant to human lung cancer. Sci. Rep..

[6] Sonnenfeld G., Butel J.S., Shearer W.T. (2003). Effects of the space flight environment on the immune system. Rev. Environ. Health.

[7] Crucian B.E., Stowe R.P., Pierson D.L., Sams C.F. (2008). Immune system dysregulation following short-vs longduration spaceflight. Aviat. Space Environ. Med..

[8] von Sonntag C. (1987). The Chemical Basis of Radiation Biology.

[9] Alwood J.S., Tran L.H., Schreurs A.S., Shirazi-Fard Y., Kumar A., Hilton D., Tahimic C.G.T., Globus R.K. (2017). Dose- and ion-dependent effects in the oxidative stress response to space-like radiation exposure in the skeletal system. Int. J. Mol. Sci..

[10] Guan J., Wan X.S., Zhou Z., Ware J., Donahue J.J., Biaglow J.E., Kennedy A.R. (2004). Effects of dietary supplements on space radiation-induced oxidative stress in Sprague-Dawley rats. Radiat. Res..

[11] Kobashigawa S., Suzuki K., Yamashita S. (2011). Ionizing radiation accelerates Drp1-dependent mitochondrial fission, which involves delayed mitochondrial reactive oxygen species production in normal human fibroblast-like cells. Biochem. Biophys. Res. Commun..

[12] Strauss O. (2005). The retinal pigment epithelium in visual function. Physiol. Rev..

[13] Yakovleva M.A., Feldman T.B., Lyakhova K.N., Utina D.M., Kolesnikova I.A., Vinogradova Y.V., Molokanov A.G., Ostrovsky M.A. (2022). Ionized radiation-mediated retinoid oxidation in the retina and retinal pigment epithelium of the murine eye. Radiat. Res..

[14] Gorgels T.G., van der Pluijm I., Brandt R.M., Garinis G.A., van Steeg H., van den Aardweg G., Jansen G.H., Ruijter J.M., Bergen A.A., van Norren D. (2007). Retinal degeneration and ionizing radiation hypersensitivity in a mouse model for Cockayne syndrome. Mol. Cell. Biol..

[15] Ponomarev A.L., Nounu H.N., Hussein H.F., Kim M.-H.Y., Atwell W., Cucinotta F.A. (2007). NASA-Developed ProE-Based Tool for the Ray-Tracing of Spacecraft Geometry to Determine Radiation Doses and Particle Fluxes in Habitable Areas of Spacecraft and in the Human Body.

[16] Cucinotta F.A., Manuel F.K., Jones J., Iszard G., Murrey J., Djojonegro B., Wear M. (2001). Space radiation and cataracts in astronauts. Radiat. Res..

[17] Mao X.W., Boerma M., Rodriguez D., Campbell-Beachler M., Jones T., Stanbouly S., Sridharan V., Wroe A., Nelson G.A. (2018). Acute effect of low-dose space radiation on mouse retina and retinal endothelial cells. Radiat. Res..

[18] Mao X.W., Archambeau J.O., Kubinova L., Boyle S., Petersen G., Grove R. (2003). Quantification of rat retinal growth and vascular population changes after single and split doses of proton irradiation: Translational study using stereology methods. Radiat. Res..

[19] Mao X.W., Pecaut M.J., Stodieck L.S., Ferguson V.L., Bateman T.A., Bouxsein M., Jones T.A., Moldovan M., Cunningham C.E., Chieu J. (2013). Space flight environment induces mitochondrial oxidative damage in ocular tissue. Radiat. Res..

[20] Mao X.W., Favre C., Fike J.R., Kubinova L., Anderson E., Campbell-Beachler M., Jones T., Smith A., Rightnar S., Nelson G.A. (2010). High-LET radiation-induced response of microvessels in the hippocampus. Radiat. Res..

[21] van Reyk D.M., Gillies M.C., Davies M.J. (2003). The retina: Oxidative stress and diabetes. Redox Rep..

[22] Yakovleva M.A., Dontsov A.E., Trofimova N.N., Sakina N.L., Kononikhin A.S., Aybush A.V., Feldman T.B., Ostrovsky M.A. (2022). Lipofuscin granule bisretinoid oxidation in the human retinal pigment epithelium forms cytotoxic carbonyls. Int. J. Mol. Sci..

[23] Dontsov A., Yakovleva M., Trofimova N., Sakina N., Gulin A., Aybush A., Gostev F., Vasin A., Feldman T., Ostrovsky M. (2022). Water-soluble products of photooxidative destruction of the bisretinoid A2E cause proteins modification in the dark. Int. J. Mol. Sci..

[24] Feldman T., Ostrovskiy D., Yakovleva M., Dontsov A., Borzenok S., Ostrovsky M. (2022). Lipofuscin-mediated photic stress induces a dark toxic effect on ARPE-19 cells. Int. J. Mol. Sci..

[25] Feldman T.B., Yakovleva M.A., Arbukhanova P.M., Borzenok S.A., Kononikhin A.S., Popov I.A., Nikolaev E.N., Ostrovsky M.A. (2015). Changes in spectral properties and composition of lipofuscin fluorophores from human retinal pigment epithelium with age and pathology. Anal. Bioanal. Chem..

[26] Wu Y., Yanase E., Feng X., Siegel M.M., Sparrow J.R. (2010). Structural characterization of bisretinoid A2E photocleavage products and implications for age-related macular degeneration. Proc. Natl. Acad. Sci. USA.

[27] Ben-Shabat S., Itagaki Y., Jockusch S., Sparrow J.R., Turro N.J., Nakanishi K. (2002). Formation of a nona-oxirane from A2E, alipofuscin fluorophore related to macular degeneration, and evidence of singlet oxygen involvement. Angew. Chem. Int. Ed..

[28] Yoon K.D., Yamamoto K., Ueda K., Zhou J., Sparrow J.R. (2012). A novel source of methylglyoxal and glyoxal in retina: Implications for age-related macular degeneration. PLoS ONE.

[29] Ergin V., Ebrahimi R., Karasu C. (2013). Carbonyl stress in aging process: Role of vitamins and phytochemicals as redox regulators. Aging Dis..

[30] Schleicher E.D., Bierhaus A., Haring H.U., Nawroth P.P., Lehmann R. (2001). Chemistry and pathobiology of advanced glycation end products. Contrib. Nephrol..

[31] Feldman T.B., Yakovleva M.A., Larichev A.V., Arbukhanova P.M., Radchenko A.S., Borzenok S.A., Kuzmin V.A., Ostrovsky M.A. (2018). Spectral analysis of fundus autofluorescence pattern as a tool to detect early stages of degeneration in the retina and retinal pigment epithelium. Eye.

[32] Feldman T.B., Ostrovsky M.A., Yakovleva M.A., Larichev A.V., Borzenok S.A., Arbukhanova P.M. (2017). A Method for the Early Detectionof Age-Related Macular Retinal Dystrophy. RF Patent No..

[33] Larichev A.V., Panchenko V.Y., Ostrovsky M.A., Feldman T.B. (2017). Optical Device for the Study of the Fundus to Detect Age-Related Macular Dystrophy of the Retina. RF Patent for Utility Model No..

[34] Ferdous S., Liao K.L., Gefke I.D., Summers V.R., Wu W., Donaldson K.J., Kim Y.-K., Sellers J.T., Dixon J.A., Shelton D.A. (2021). Age-related retinal changes in wild-type C57BL/6J mice between 2 and 32 months. Investig. Ophthalmol. Vis. Sci..

[35] Sparrow J.R., Wu Y., Nagasaki T., Yoon K.D., Yamamoto K., Zhou J. (2010). Fundus autofluorescence and the bisretinoids of retina. Photochem. Photobiol. Sci..

[36] Kim S.R., Jang Y.P., Jockusch S., Fishkin N.E., Turro N.J., Sparrow J.R. (2007). The all-*trans*-retinal dimer series of lipofuscin pigments in retinal pigment epithelial cells in a recessive Stargardt disease model. Proc. Natl. Acad. Sci. USA.

[37] Azzam E.I., Jay-Gerin J.P., Pain D. (2012). Ionizing radiation-induced metabolic oxidative stress and prolonged cell injury. Cancer Lett..

[38] Belli M., Indovina L. (2020). The response of living organisms to low radiation environment and its implications in radiation protection. Front. Public Health.

[39] Chien T., Tseng T.L., Wang J.Y., Shen Y.T., Lin T.H., Shieh J.C. (2015). Candida albicans DBF4 gene inducibly duplicated by the mini-Urablaster is involved in hypha-suppression. Mutat. Res..

[40] Saha A.K., Kappes F., Mundade A., Deutzmann A., Rosmarin D.M., Legendre M., Chatain N., Al-Obaidi Z., Adams B.S., Ploegh H.L. (2013). Intercellular trafficking of the nuclear oncoprotein DEK. Proc. Natl. Acad. Sci. USA.

[41] Kennedy C.J., Rakoczy P.E., Constable I.J. (1995). Lipofuscin of the retinal pigment epithelium: A review. Eye.

[42] Amoaku W.M., Mahon G.J., Gardiner T.A., Frew L., Archer D.B. (1992). Late ultrastructural changes in the retina of the rat following low-dose X-irradiation. Graefe’s Arch. Clin. Exp. Ophthalmol..

[43] Rózanowska M., Sarna T. (2005). Light-induced damage to the retina: Role of rhodopsin chromophore revisited. Photochem. Photobiol..

[44] Feldman T.B., Dontsov A.E., Yakovleva M.A., Ostrovsky M.A. (2022). Photobiology of lipofuscin granules in the retinal pigment epithelium cells of the eye: Norm, pathology, age. Biophys. Rev..

[45] Kubančák J., Molokanov A.G. (2013). Measurements of LET spectra of the JINR phasotron radiotherapy proton beam. Probl. At. Sci. Technol..

[46] Mata N.L., Weng J., Travis G.H. (2000). Biosynthesis of a major lipofuscin fluorophore in mice and humans with ABCR-mediated retinal and macular degeneration. Proc. Natl. Acad. Sci. USA.

[47] Folch J., Lees M., Stanley G.H.S. (1957). A simple method for the isolation and purification of total lipides from animal tissues. J. Biol. Chem..

[48] Holz F.G., Fleckenstein M., Schmitz-Valckenberg S., Bird A.C., Holz F.G., Schmitz-Valckenberg S., Spaide R.F., Bird A.C. (2007). Evaluation of fundus autofluorescence images. Atlas of Fundus Autofluorescence Imaging.

[49] Parish C.A., Hashimoto M., Nakanishi K., Dillon J., Sparrow J. (1998). Isolation and one-step preparation of A2E and iso-A2E, fluorophores from human retinal pigment epithelium. Proc. Natl. Acad. Sci. USA.

